# Reduced crown root number improves water acquisition under water deficit stress in maize (*Zea mays* L.)

**DOI:** 10.1093/jxb/erw243

**Published:** 2016-07-08

**Authors:** Yingzhi Gao, Jonathan P. Lynch

**Affiliations:** ^1^Key Laboratory of Vegetation Ecology, Institute of Grassland Science, Northeast Normal University, Changchun 130024, Jilin Province, China; ^2^Department of Plant Science, Pennsylvania State University, University Park, PA 16802, USA

**Keywords:** Crown root number, drought, maize, respiration, rooting depth, water stress.

## Abstract

In this study we test the hypothesis that maize genotypes with reduced crown root number (CN) will have greater root depth and improved water acquisition from drying soil. Maize recombinant inbred lines with contrasting CN were evaluated under water stress in greenhouse mesocosms and field rainout shelters. CN varied from 25 to 62 among genotypes. Under water stress in the mesocosms, genotypes with low CN had 31% fewer crown roots, 30% deeper rooting, 56% greater stomatal conductance, 45% greater leaf CO_2_ assimilation, 61% net canopy CO_2_ assimilation, and 55% greater shoot biomass than genotypes with high CN at 35 days after planting. Under water stress in the field, genotypes with low CN had 21% fewer crown roots, 41% deeper rooting, 48% lighter stem water oxygen isotope enrichment (δ^18^O) signature signifying deeper water capture, 13% greater leaf relative water content, 33% greater shoot biomass at anthesis, and 57% greater yield than genotypes with high CN. These results support the hypothesis that low CN improves drought tolerance by increasing rooting depth and water acquisition from the subsoil.

## Highlight

Maize genotypes with reduced crown root number have superior water capture, growth, and yield under drought.

## Introduction

The identification and understanding of root phenes improving drought tolerance are essential for the development of more drought-tolerant crops (Lynch, 2014). Plants have evolved a variety of mechanisms to adapt to water stress (Javot and Maurel, 2002; Deak and Malamy, 2005; Nord and Lynch, 2009; Gao *et al.*, 2013), e.g. shedding leaves to reduce transpiration; modifying aquaporin (AQP) expression to reduce hydraulic conductivity; osmotic adjustment; and adjusting phenology to avoid drought events. During the development of terminal drought, soil dries from the top of the profile exposing the upper part of the root system to water stress, while deeper roots may still be able to access deeper soil water for plant growth. Deeper rooting is an important way to access water stored in deep soil (Lynch, 2007, 2013; Lynch *et al.*, 2014; Lynch and Wojciechowski, 2015). In recent years several studies have demonstrated that root phenes and phene states that reduce the metabolic cost of soil exploration permit greater root growth, which improves the capture of deep soil resources including nitrate and water (Zhu *et al.*, 2010; York *et al.*, 2013; Chimungu *et al.*, 2014*a*, *b*; Lynch, 2014; Zhan and Lynch, 2015; Zhan *et al.*, 2015).

An ideotype has been proposed to guide the breeding of crops with deeper roots and greater water acquisition from drying soil, called ‘Steep, Cheap, and Deep’ (SCD), integrating architectural, anatomical, and physiological phenes (Lynch, 2013). ‘Cheap’ refers to phenes that reduce the metabolic cost of soil exploration, including root anatomical phenes such as decreased cortical cell file number and increased cortical cell size (Chimungu *et al.*, 2014*a*, *b*), or increased formation of root cortical aerenchyma (Zhu *et al.*, 2010; Saengwilai *et al.*, 2014*a*; Chimungu *et al.*, 2015). ‘Steep’ refers to architectural phenes capable of positioning root foraging in deeper soil domains, either directly via for example root growth angle (Trachsel *et al.*, 2011; Burton *et al.*, 2014) or by focusing plant investment in elongation of axial roots (Saengwilai *et al.*, 2014*b*). Two phenes proposed for this latter function are reduced number of axial roots and reduced lateral branching density. Both modeling (Postma *et al.*, 2014) and empirical results from the field (Zhan and Lynch, 2015) affirm the value of reduced lateral root branching in the capture of nitrate by maize. Reduced lateral root branching density can improve drought tolerance in maize by reducing the metabolic costs of soil exploration, permitting greater axial root elongation, greater rooting depth, and thereby greater water acquisition from drying soil (Zhan *et al.*, 2015). Reduced axial root formation benefits nitrate capture in maize, probably due to reduced competition among roots of the same plant for both internal and external resources (Saengwilai *et al.*, 2014*b*). A recent comparison of leading maize lines over the past century associated reduced formation of axial roots (crown roots in maize) with improved N capture (York *et al.*, 2015). However, the utility of reduced axial root formation for water capture from drying soil has not been tested, and is the focus of this study.

The production of axial roots is a key element of root phenotypes. Axial roots are major structural investments of root biomass and form the primary structural framework from which lateral roots emerge. The location of axial roots in the soil has an important influence on the foraging of soil domains by lateral roots and root symbionts. The production of a large number of axial roots could be counterproductive by increasing the spatial proximity of root foraging and therefore intraplant competition for soil resources, and also by increasing internal resource competition among competing root sinks, resulting in, for example, reduced lateral root development or reduced elongation of axial roots (Lynch, 2014; Saengwilai *et al.*, 2014*b*). On the other hand production of a small number of axial roots may decrease physical support of the shoot, decrease the intensity of soil exploration, increase the risk of loss of root function via herbivory and soil pathogens, and decrease the ability of a plant to compete with its neighbors for soil resources. The SCD ideotype proposes that axial root number be optimized so that these constraints are balanced, i.e. sufficient axial roots are produced to permit adequate soil exploration, but beyond this number axial root production would be counterproductive for the capture of N and water (Lynch, 2013).

Maize (*Zea mays* L.) is the leading global crop, and in Africa and Latin America is an important staple food (Grassini *et al.*, 2013). The maize root system is composed of a primary root, a variable number of seminal roots, nodal roots arising from belowground stem nodes (crown roots) and aboveground stem nodes (brace roots), and lateral roots arising from these axes (Hochholdinger *et al.*, 2004). Crown root number (CN), consisting of the number of belowground nodal whorls and the number of roots per whorl, is a central feature of maize root architecture (Saengwilai *et al.*, 2014*b*). The crown root system dominates resource acquisition during vegetative growth after the first few weeks and remains important during reproductive development (Hoppe *et al.*, 1986; Hochholdinger *et al.*, 2004; Yu *et al.*, 2014). There is substantial genotypic variation for CN in maize, varying from five to 75 (Bayuelo-Jiménez *et al.*, 2011; Gaudin *et al.*, 2011; Trachsel *et al.*, 2011; Burton *et al.*, 2014; Saengwilai *et al.*, 2014*b*; York *et al.*, 2015).

The objective of this study was to test the hypothesis that maize genotypes with reduced CN will have greater rooting depth, and therefore better water acquisition from subsoil strata under water stress, resulting in better plant water status, growth and yield.

## Materials and methods

### Plant materials

Eight genotypes from three recombinant inbred line (RIL) populations were selected, RILs IBM 009 and 123 from the intermated population of B73×Mo17 (Sharopova *et al.*, 2002), OHW 74 and 170 from Oh43×W64a (OhW), and NYH 41, 51, 57, and 224 from Ny821×H99 (Burton *et al.*, 2014). In previous studies these genotypes had contrasting CN (Burton *et al.*, 2014; Saengwilai *et al.*, 2014*a*). All seeds were obtained from Shawn Kaeppler, University of Wisconsin, Madison, WI, USA.

### Greenhouse mesocosm experiment

A 2×8 factorial randomized complete block design was carried out in a greenhouse. Factors were two water regimes and eight genotypes. Four replicates were staggered 7 days between replicates with time of planting treated as a block effect.

Seeds were surface-sterilized in 0.05% NaOCl for 15min and imbibed for 24h in aerated 1mM CaSO4, then placed in darkness at 25 °C for 2 days. Seedlings were transplanted to mesocosms consisting of PVC cylinders 0.15 m×1.5 m lined with 4 mil (0.116mm) transparent hi-density polyethylene film. The growth medium was (v/v) 50% commercial grade sand, 35% #3 vermiculite, 5% perlite, and 10% sieved topsoil. The soil was a Typic Hapludalf, pH 6.7, silt loam. Nutrients were supplied by 70g per column of Osmocote Plus fertilizer consisting of (%): N (15), P (9), K (12), S (2.3), B (0.02), Cu (0.05), Fe (0.68), Mn (0.06), Mo (0.02), and Zn (0.05) (Scotts-Sierra Horticultural Products Company, Marysville, OH, USA). Twenty-nine liters of medium was used in each cylinder. One day before planting, cylinders were given 4.5L deionized water. Each cylinder received three plants; after 5 d they were thinned to one plant. Plants were grown in a temperature-controlled greenhouse in University Park, PA, USA (40°49′N, 77°49′W), with a photoperiod of 14/10h at 28/24 °C (light/darkness), 1200 µmol photons m^−2^ s^−1^ maximum PAR, and 40–70% relative humidity. Plants received 100ml of water every day for 4 d, then 250ml of water was applied to the WW treatment every 2 d. In the water stress treatment, there was no further irrigation.

#### Leaf net photosynthesis rate, canopy photosynthesis and total root respiration

Plants were harvested from mesocosms 5 weeks after transplanting. Four days before harvest, leaf gas exchange of the youngest fully expanded leaf was measured with a Licor-6400 infrared gas analyser (Li-Cor Biosciences, Lincoln, NE, USA) 1200 μmol photons m^−2^ s^−1^ PAR, 400ppm CO_2_, 25 °C leaf temperature, and 40% relative humidity. Canopy photosynthesis was measured 32 d after planting (DAP) with a Li-Cor 6400. A 37.6L (30×28×45cm) transparent acrylic chamber enclosed the whole shoot as described in Jaramillo *et al.* (2013). For measurement of intact root respiration we used the ‘head space’ approach 2 d before harvest (Nielsen *et al.*, 1998). The measurements were conducted in the morning with a Li-6400. Each measurement required 2–4min. We assume that microbial respiration was comparable among cylinders (Bouma *et al.*, 1997). Intact root system respiration was divided by the total root length obtained by WinRhizo scanning to obtain the specific root respiration per unit of root length.

#### Leaf relative water content

To measure leaf relative water content (LRWC), fresh leaf discs (3cm diameter) were collected from the third fully expanded leaf at 34 DAP and weighed immediately to determine fresh weight (FW), after which the discs were hydrated to full turgidity (6h) by soaking them in distilled water. Following soaking, the discs were blotted dry and again weighed to determine turgid weight (TW). Discs were then dried at 70 °C for 72h, and dry weight (DW) was determined. LRWC was calculated according to the equation: LRWC (%)=100×(FW–DW)/(TW–DW).

#### Water ^18^O injection and stem base sampling

The ability of roots to acquire water from deep soil strata was studied by deep injection H_2_^18^O-labeled water (97 atom%). Three holes were made at 90cm depth in each cylinder, 3mL of labelled water (water H_2_^18^O 97 atom%, 0.5mg ml^−1^) was injected into the tube in each hole. Following the injection each hole was sealed with adhesive putty. The injections were made at 34 DAP, and plants were harvested 16–18h after injection. At harvest, 35 DAP, the shoot was cut at the stem base, segments of stem were cut into 8–10cm lengths, placed into 40ml vials in dry ice, then transferred to –20 °C. The rest of the shoot was dried at 70 °C for 72h for biomass.

#### Soil water content and root length distribution

Samples for soil water measurements were collected at 20cm depth increments before washing. Soil water content is presented for a single time point in the mesocosm study because of the shorter duration of this study and the difficulty of accurately measuring soil water content in small volumes. Samples were dried at 75 °C for 80h for determination of soil water content (%SWC=(soil weight with water–soil dry weight)/soil dry weight*×*100%). The roots were extracted by rinsing the media with water. Roots from each 20cm depth increment were spread in a 3–5mm layer of water in transparent Plexiglas trays and imaged with a flatbed scanner equipped with top lighting (Epson Perfection V700 Photo, Epson America, Inc., USA) at a resolution of 23.6 pixels mm^−1^ (600 dpi). Root length was quantified using WinRhizo Pro (Regent Instruments, Québec City, Québec, Canada), then dried at 75 °C for 80h for biomass measurement. To summarize root distribution we used *D*_95_ (Trachsel *et al.*, 2013), i.e. the depth above which 95% of the root length is located.

### Field experiment

#### Experimental design and growth conditions

The field experiment was conducted at the Russell E. Larson Experimental Farm of The Pennsylvania State University (40°43′N, 77°56′W). A randomized complete block design with a split-plot arrangement of treatments was employed. There were four biological replicates for each of eight genotypes, employing the same genotypes used in the mesocosm studies. The experiment was planted on 25 May 2014 and each replicate had 30 plants grown in three rows with 0.76 m inter-row spacing and 0.23 m in-row spacing, resulting in a plant population of 57 000 plants ha^−1^. The shelters (10×30 m) were covered with a clear polyethylene film and were automatically triggered by rainfall to cover the plots, excluding natural precipitation from 10 May to 25 September. Adjacent non-sheltered plots were drip-irrigated as needed to provide unstressed comparisons. The soil was a Murrill silt loam (fine-loamy, mixed, semiactive, mesic Typic Hapludult).

#### Soil water content and leaf relative water content

Soil volumetric water content was monitored using a TRIME FM system (IMKO Micromodultechnik GmbH, Ettlingen, Germany) at three depths (15, 30 and 50cm) both inside and outside the rainout shelters. In each plot, two TRIME FM systems were installed along the maize row. Fifteen readings from each monitoring system were taken between 24 and 127 DAP.

#### Net photosynthesis rate and leaf relative water content

Four days before harvest, net photosynthesis rate (Pn) was recorded on the ear leaf. Pn was measured as described above but at 1800 μmol photons m^−2^ s^−1^, 400ppm CO_2_, 25 °C leaf temperature, and 40% relative humidity. LRWC was measured as described above, except that nine fresh leaf discs were collected from the ear leaf for three representative plants per plot.

#### Shoot biomass, crown root number, biomass, and grain yield

Plants were harvested at anthesis (80 DAP). Three adjacent plants were randomly selected in the same row for shoot dry weight. Roots were excavated by removing a soil cylinder *ca* 40cm diameter and 25cm depth from the plant stem. Excavated root crowns were rinsed followed by manual quantification of CN. At physiological maturity (127 DAP), grain yield was collected. All samples were oven-dried at 75 °C for 100h for biomass.

#### Rooting depth

Soil cores were collected 80 DAP. A soil coring tube (5.1 cm×60cm) was placed midway between plants within a row. Cores were sectioned into 10cm increments and washed. Washed roots were scanned (Epson, Perfection V700 Photo) at a resolution of 23.6 pixels mm^–1^ (600 dpi) and analysed using WinRhizo Pro. Root distribution was calculated as described above, and roots were dried at 70 °C for 80h for biomass.

#### Soil and shoot xylem (δ^18^O)

Soil was sampled 3–5cm from plants in the rainout shelter 80 DAP with a 5cm diameter core to 60cm depth and separated into 10cm increments. Approximately 8–10cm of the associated stem was collected just above the soil surface and the epidermis was immediately removed. Soil and stem samples were placed in 40ml vials, sealed with parafilm, placed in dry ice, then stored at –20 °C. Soil and stem water was extracted with cryogenic vacuum distillation (Koeniger *et al.*, 2010; Chimungu *et al.*, 2014*a*) and analysed using a Picarro L2130-i δD/δ^18^O ultra high precision isotopic water analyser (Picarro Inc., CA, USA) at the Natural Resource Ecology Laboratory, Colorado State University. Results were expressed as parts per thousand deviations from the Vienna Standard Mean Ocean Water (VSMOW). IsoSource version 1.3.1 (Phillips and Gregg, 2003; Phillips *et al.*, 2005) was used to evaluate the relative contribution of each soil layer to plant water signature. The fractional increment was set at 1%, and tolerance at 0.1.

### Data analysis

Statistical analyses employed SPSS 17.0 (SPSS Inc., Chicago, IL, USA). Two-way ANOVA was used to assess the effects of high- and low-CN lines, water, and their interaction. Tukey’s HSD test was used for multiple comparisons. Differences of soil water content in the same soil depth between water-stressed (WS) and well-watered (WW) treatments and root length density in the same soil depth between high CN and low CN phenotypes were analysed by *t*-test. Linear regression analysis and Pearson correlation coefficients were calculated using Sigmaplot (Systat Software Inc., CA, USA). Significance level was set at *P*≤0.05.

## Results

### Water stress effects on soil water content

Mesocosms were used to generate stratified water distribution. Soil water content (g g^−1^ dry soil, %) in well-watered treatments was significantly greater than in water stressed treatments to 60–80cm depth at 35 DAP (see Supplementary Fig. S1 at *JXB* online). At 0–20cm, soil water content under water stress was less than 10%, only about half the amount of well-watered treatments, and water content gradually increased with increasing depth (Supplementary Fig. S1). In the field, volumetric soil moisture (m^3^ m^−3^) ranged from 25 to 38% at 10cm depth, from 30 to 38% at 30cm, and from 25 to 36% at 50cm in well-watered conditions throughout the season (Supplementary Fig. S2). For water-stress treatments, soil moisture progressively decreased from 30 to 13% at 10cm, from 22 to 15% at 30cm, and remained stable from 18 to 22% at 50cm (Supplementary Fig. S2).

### Water stress effects on crown root number

**Fig. 1.**
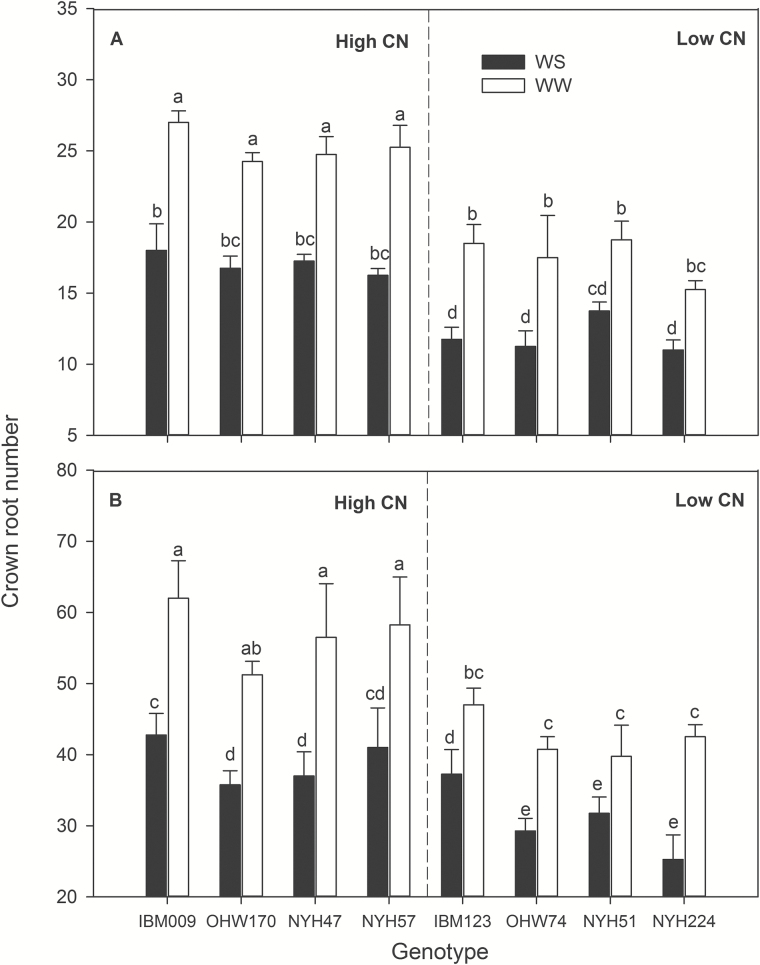
Crown root number (CN) of maize 35 DAP in greenhouse mesocosms (A) and at anthesis in the field (B) under water-stressed (WS) and well-watered (WW) conditions. Bars show means of four replicates+SE. Different letters represent significant differences between means within the same section (*P*<0.05).

**Fig. 2.**
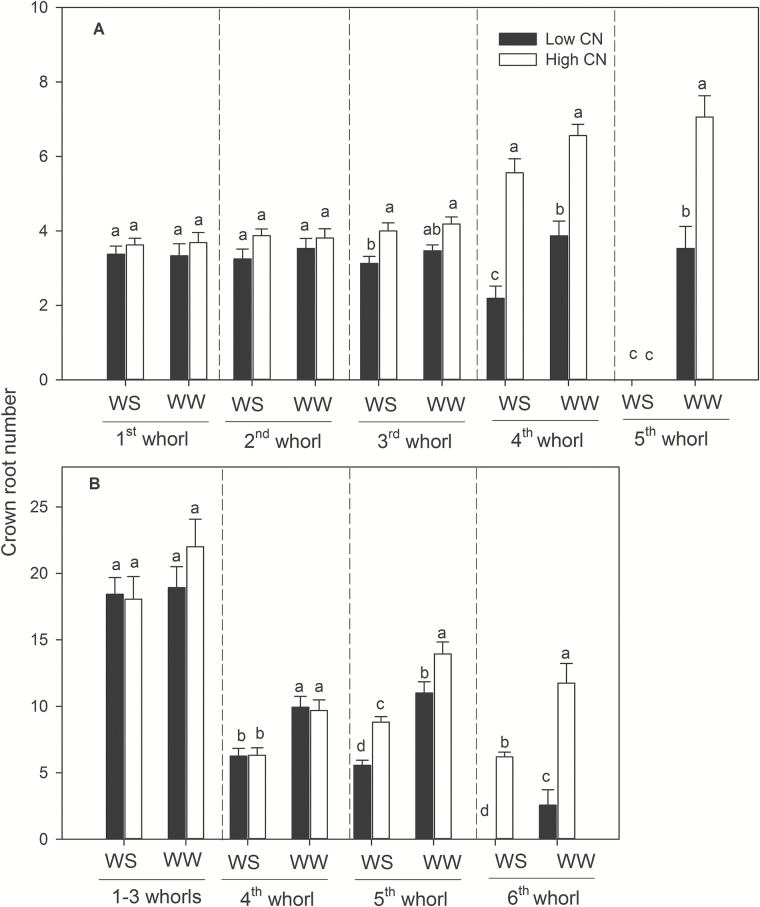
Crown root number per whorl of maize 35 DAP in greenhouse mesocosms (A) and at anthesis in the field (B) under water-stressed (WS) and well-watered (WW) conditions. The data shown are means of four replicates of the four genotypes (+SE) in each phenotypic class of either high CN or low CN. Different letters represent significant differences (*P*<0.05) compared within each root whorl.

In well-watered plants, CN was greater in ‘high CN’ than in ‘low CN’ categories in both mesocosms and field conditions (Fig. 1). In mesocosms, water stress significantly decreased CN for all genotypes, by an average of 28% at 35 DAP (Fig. 1 and Supplementary Table S1). Under water stress, the CN varied from 11 to 18, and was significantly less in low-CN genotypes than in high-CN genotypes, except NYH51. The intermediate CN phenotypes of NYH51 and IBM123 did not substantially affect results: whether these genotypes were classified as having low CN, high CN, or were excluded entirely from the analyses, category means for low CN and high CN phenotypes under water stress were comparable for CO_2_ assimilation rate, stomatal conductance, LRWC, net canopy CO_2_ assimilation, total root respiration, crown root number and *D*_95_ (see Supplementary Table S2). Water stress did not influence the number of crown roots in the first, second and third nodes but significantly reduced the number of axial roots of the fourth and fifth nodes, and there was no fifth node development for low-CN genotypes under water stress (Fig. 2). Low-CN genotypes had fewer nodes than high-CN genotypes. In the field, water stress reduced CN by an average of 30% at flowering. Under water stress, the CN ranged from 25 to 43, and CN remained significantly greater in high-CN genotypes than in low-CN genotypes, except for IBM123 (Fig. 1 and Supplementary Table S3). Water stress did not affect the number of roots in the first, second and third nodes but significantly decreased the number of roots of the fourth, fifth and sixth nodes, particularly in low-CN genotypes (Fig. 2). Low-CN genotypes had significantly fewer roots in the fifth node than high-CN genotypes and had no sixth node development under water stress.

### CN effects on photosynthesis and total root respiration

**Fig. 3.**
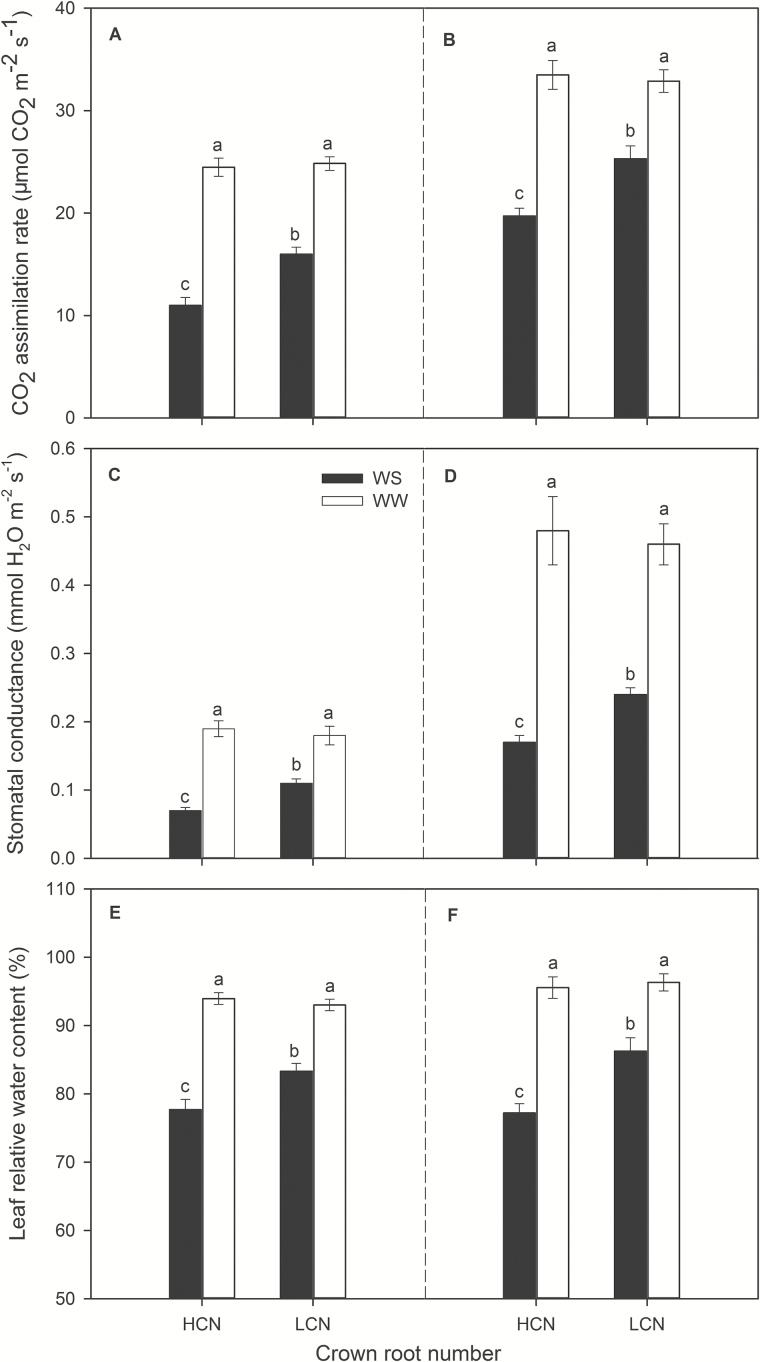
Leaf CO_2_ assimilation rate (μmol CO_2_ m^−2^ s^−1^), leaf stomatal conductance (mmol H_2_O m^−2^ s^−1^) and leaf relative water content (% w/w) at 35 DAP in greenhouse mesocosms (A, C, E), at anthesis in the field (B, D, F) under water-stressed and well-watered conditions. The data shown are means of four replicates for each of four genotypes in each phenotype category±SE. Different letters represent significant differences within a panel at the level of α=0.05. HCN: high CN; LCN: low CN.

**Fig. 4.**
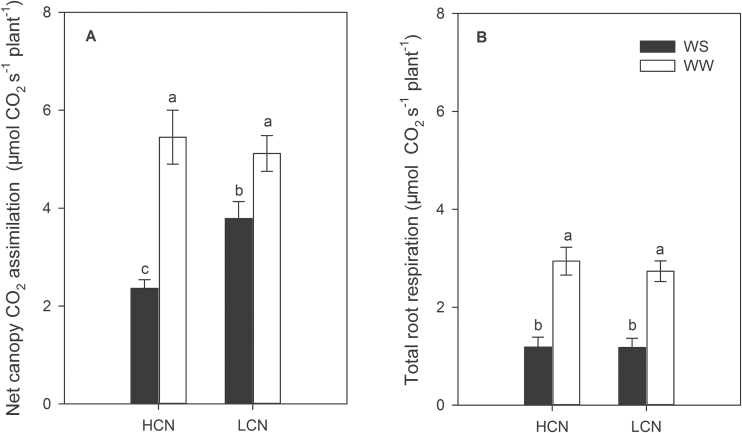
Net canopy CO_2_ assimilation (μmol CO_2_ s^−1^ plant^−1^) (A) and total root respiration (μmol CO_2_ s^−1^ plant^−1^) (B) at 35 DAP in greenhouse mesocosms under water-stressed and well-watered conditions. The data shown are means of four replicates for each of four genotypes in each phenotype category±SE. Different letters represent significant differences within each panel at the level of α=0.05. HCN: high CN; LCN: low CN.

Water availability and genotype affected leaf CO_2_ assimilation rate, stomatal conductance, and canopy photosynthesis (Figs 3 and 4 and Supplementary Tables S1 and S3). Under water stress, genotypes with low CN had 45% (greenhouse) and 32% (field) greater leaf photosynthesis, 56% (greenhouse) and 40% (field) greater stomatal conductance, and 61% (greenhouse) greater canopy photosynthesis than genotypes with high CN. However, there was no significant difference in leaf and canopy photosynthesis and stomatal conductance between high-CN and low-CN genotypes under well-watered conditions. In addition, water stress significantly reduced total root respiration by an average of 58%, regardless of CN phenotype (Fig. 4 and Supplementary Table S1). Similarly, specific root respiration (pmol CO_2_ cm^−1^ s^−1^) was not different between high-CN and low-CN phenotypes, but water stress reduced specific root respiration by an average of 46% (Supplementary Fig. S3 and Supplementary Table S1).

### CN effects on rooting depth and water acquisition

**Fig. 5.**
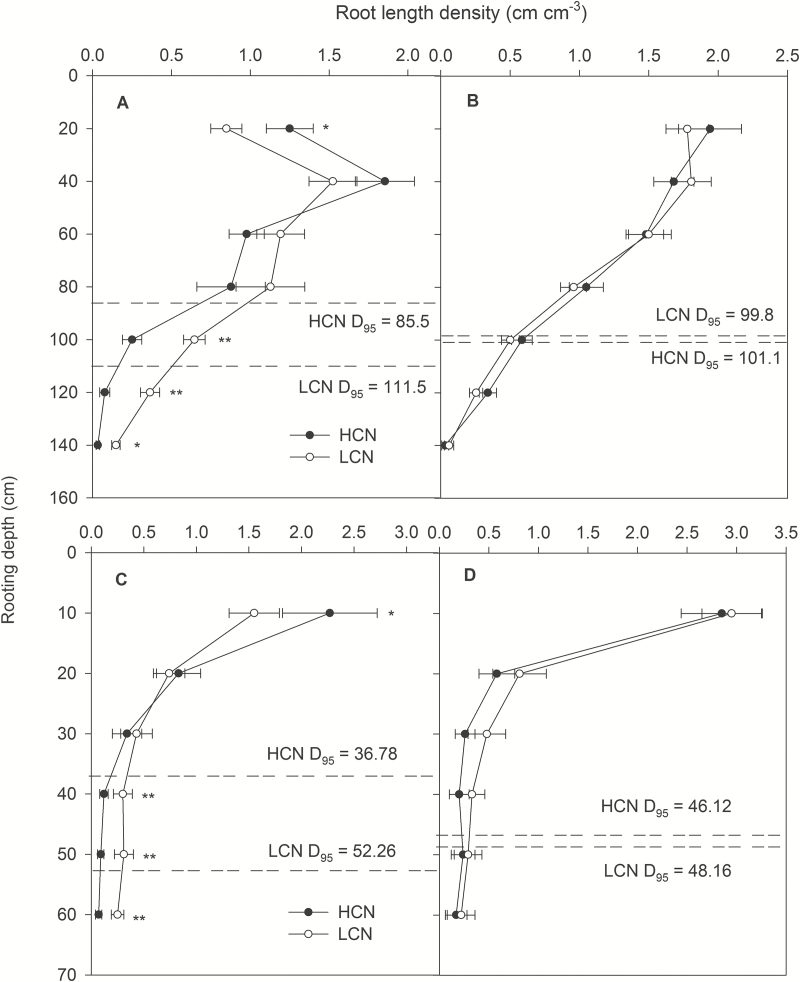
Root length density (cm cm^−3^) of maize at 35 DAP in greenhouse mesocosms under water stress (A) and well-watered (B) conditions, and at anthesis in the field under water stress (C) and well-watered (D) conditions. The data shown are the mean of four replicates of the four genotypes of high CN and low CN (±SE). The average values of *D*_95_ for four replicates of four high-CN and four low-CN genotypes are shown in each panel. *P<0.05, **P<0.001. HCN: high CN; LCN: low CN.

**Fig. 6.**
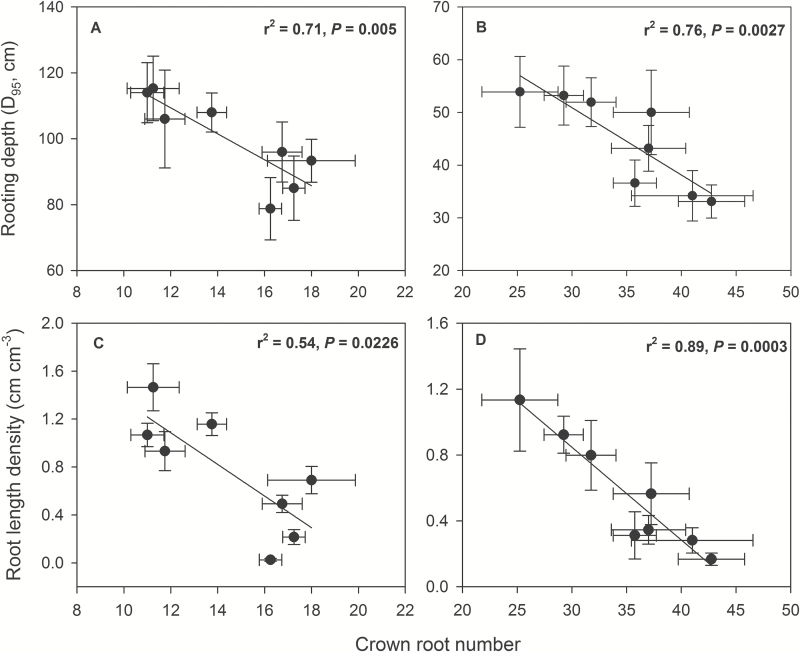
Correlations between crown root number and rooting depth (*D*_95_, cm) and root length density (cm cm^−3^) from 80–140cm soil depths of maize at 35 DAP in greenhouse mesocosms (A, C), and from 40–60cm soil depth at anthesis in the field (B, D) under water-stressed conditions. Each point is the mean of four replicates of each genotype (±SE).

Under water stress, genotypes with low CN had 30% greater rooting depth (*D*_95_, the depth above which 95% of total root length is located in the soil profile) in mesocosms and 41% greater rooting depth in the field, and 8% (greenhouse) and 13% (field) greater LRWC than genotypes with high CN (Figs 3 and 5). Patterns of root length with depth paralleled root volume with depth (see Supplementary Table S4) because root diameter was not affected by CN phenotype (Supplementary Table S5). Rooting depth under water stress was closely associated with CN in both mesocosms (*r* ^2^=0.71, *P*=0.005) and field (*r* ^2^=0.76, *P*=0.0027; Fig. 6). In addition, genotypes with deeper *D*_95_ had greater LRWC than genotypes with shallow *D*_95_, while there was no relationship in well-watered conditions (Supplementary Fig. S4). Moreover, total root length density under water stress from 80–140cm in mesocosms (*r* ^2^=0.54, *P*=0.0226), and from 40–60cm in the field (*r* ^2^=0.89, *P*=0.0003) was closely associated with CN (Fig. 6). Low-CN genotypes proliferated more roots in soil domains below 80cm in the mesocosms and below 30cm in the field compared with high-CN genotypes under water-stressed conditions (Fig. 5), while there was no significant difference in well-watered conditions (Fig. 5). Marginally significant correlations were found between CN and rooting depth (*r* ^2^=0.34, *P*=0.08 and *r* ^2^=0.10, *P*=0.2261) and root length density (*r* ^2^=0.28, *P*=0.1016 and *r* ^2^=0.31, *P*=0.0896) in deep soil for primary and seminal roots, respectively (Supplementary Fig. S5).

### Soil and stem water δ^18^O signature

**Fig. 7.**
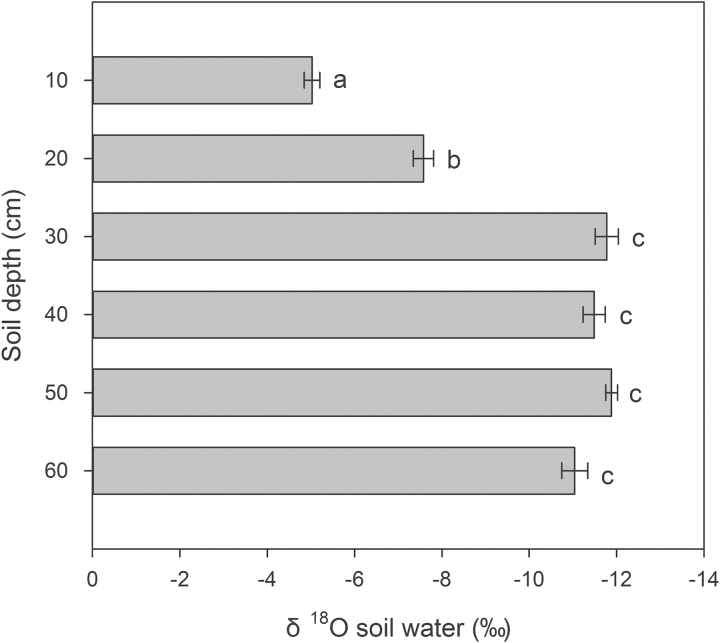
Soil water oxygen isotope composition in six soil layers in the field under water stress conditions. Values are means±SE of four observation points. Different letters represent significant differences at α=0.05.

**Fig. 8.**
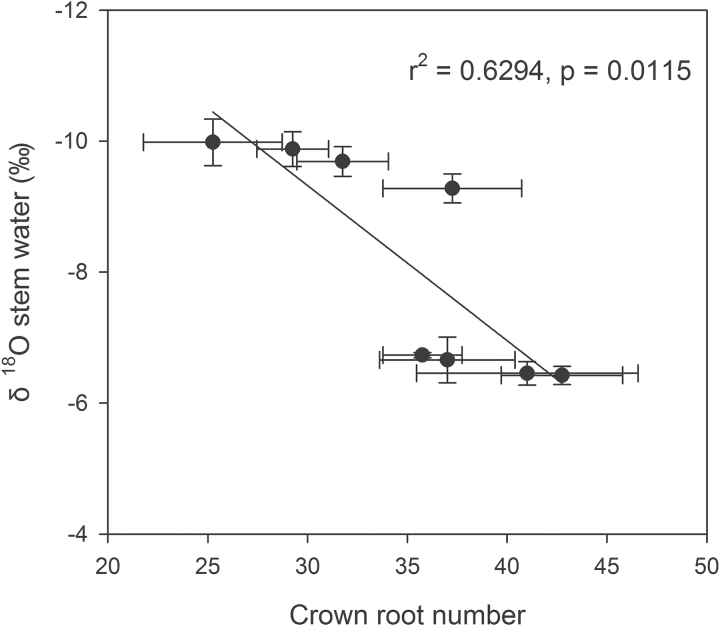
Correlation of δ^18^O of stem water and crown root number at anthesis in the rainout shelters under water stress conditions. Each point is the means ±SE of four replicates of each genotype.

Under water stress in the field, soil water δ^18^O was significantly more enriched in the upper 20cm of the soil profile and progressively declined with depth (Fig. 7), with the greatest change in the top two soil layers (averaging 3.26‰). Below 30cm soil water δ^18^O values were comparable and were aggregated as ‘deep water’ for subsequent analyses (Fig. 7). Mean values of stem water δ^18^O ranged from –9.98 to –6.42‰ (Table 1). Low CN genotypes had 48% lighter stem water signature than genotypes with high CN. An isotopic mixing model showed that low CN lines mainly absorbed ‘deep water’ (i.e. below 30cm), averaging 69% of stem water, while the high CN lines had greater dependency on the two most shallow soil layers (Table 1). CN was negatively associated with the δ^18^O signature in stem water (*r* ^2^=0.6294, *P*=0.0115; Fig. 8).

**Table 1.** Means of δ^18^O of stem water±SE measured for eight maize recombinant inbred lines (RILs) with contrasting crown root number under water stress conditions at anthesis in the rainout shelters in Pennsylvania Proportional water use by depth from different soil layers where ‘deep’ is the aggregate of three deep soil layers was calculated using multi-source mixing model analysis (Phillips *et al.*, 2005). The same letters within a column are not significantly different at the α=0.05 level. HCN: high crown root number; LCN: low crown root number.

**Classification based on CN**	**RIL**	**δ** ^**18**^ **O of stem xylem water**	**Proportional water use by depth (%**)		
			**10 cm**	**20 cm**	**Deep**
HCN	IBM009	–6.42±0.14 a	61.25	23.86	14.89
	OHW170	–6.73±0.04 a	56.35	26.78	16.87
	NYH47	–6.66±0.34 a	57.32	27.71	14.97
	NYH57	–6.45±0.18 a	60.34	25.67	13.99
LCN	IBM123	–9.28±0.22 b	11.47	21.95	66.58
	OHW74	–9.88±0.26 b	8.43	22.18	69.38
	NYH51	–9.69±0.23 b	9.16	23.59	67.25
	NYH224	–9.98±0.35 b	9.58	18.17	72.25
Mean	HCN	–6.57±0.10 a	58.82	26.01	15.18
	LCN	–9.71±0.35 b	9.66	21.47	68.87

### CN effects on shoot biomass and yield

**Fig. 9.**
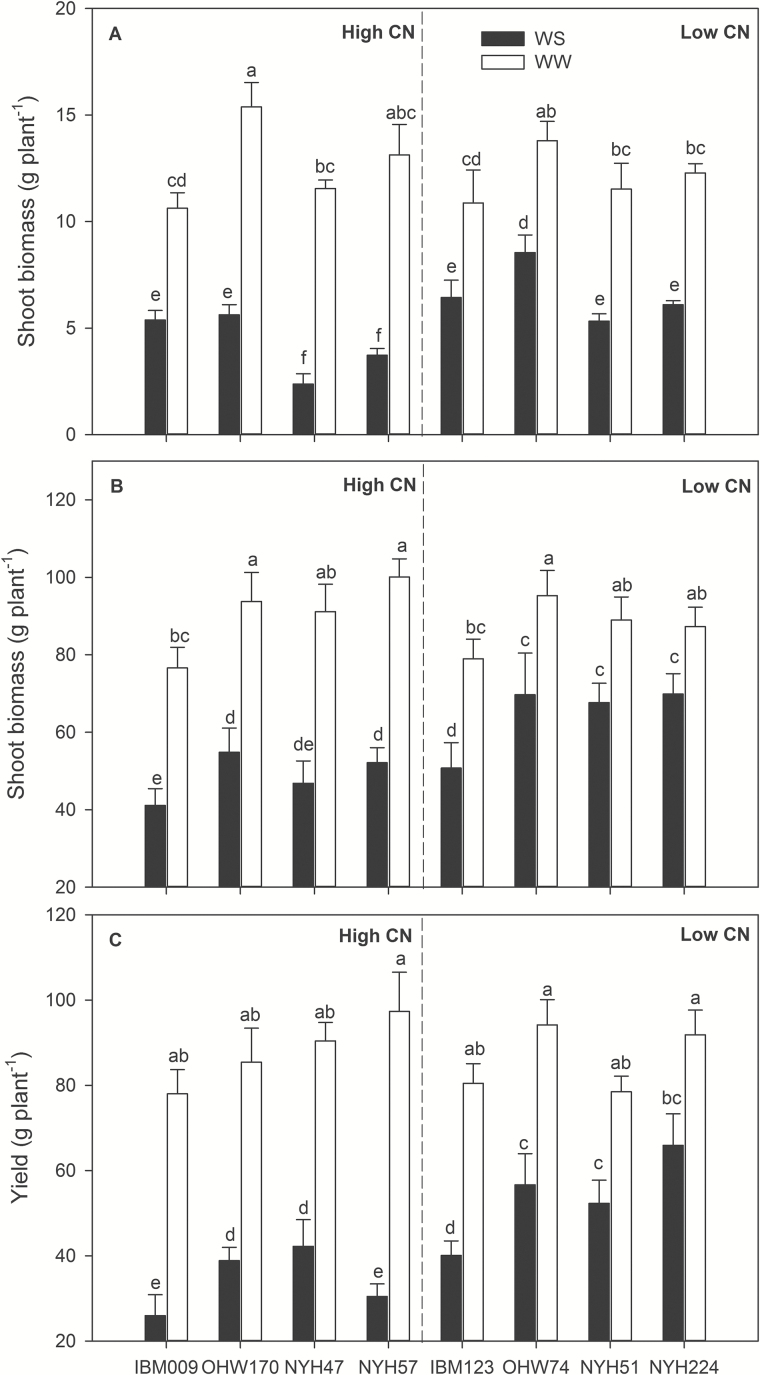
Shoot biomass (dry weight) of maize 35 DAP in greenhouse mesocosms (A), and shoot biomass (dry weight) (B) and yield (C) at anthesis in the field under water-stressed (WS) and well-watered (WW) conditions. Bars shown are means of four replicates±SE. Different letters represent significant differences among means within the three panels (*P*<0.05).

**Fig. 10.**
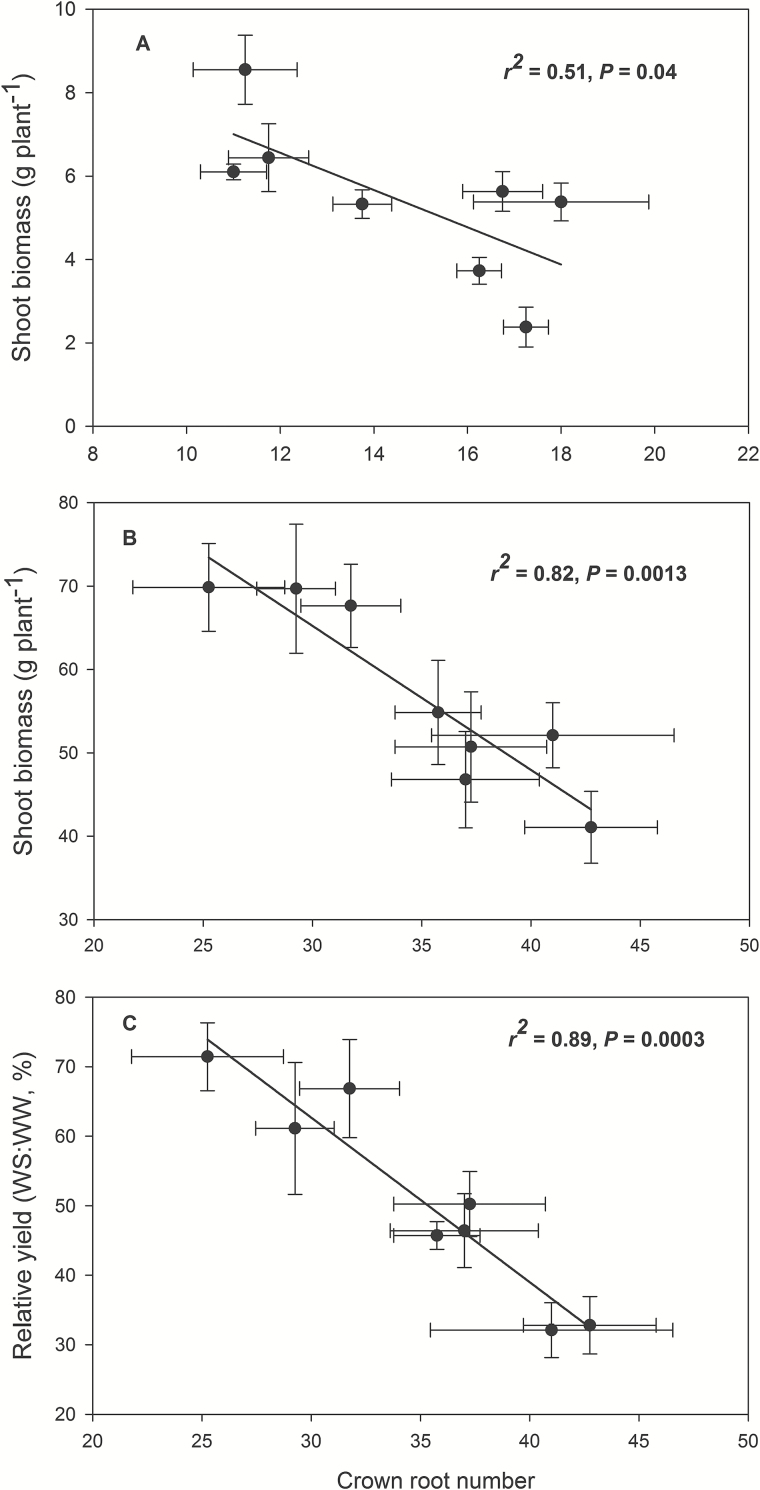
Correlations between crown root number and shoot biomass of maize at 35 DAP in greenhouse mesocosms (A), and shoot biomass (B) and relative yield (WS:WW, %) (C) at anthesis in the field under water-stressed conditions. Each point is the mean of four replicates of each genotype±SE.

Water stress in the mesocosms reduced shoot biomass at 35 DAP by 56% (Fig. 9A). The percentage of shoot biomass reduction in high-CN genotypes ranged from 49 to 79%, which was greater than the percentage reduction of low-CN genotypes, which varied from 38 to 53% (*P*<0.05). When the comparison was done within each population, shoot biomass was not significantly different between high-CN and low-CN phenotypes under well-watered conditions, while shoot biomass was reduced by 45% in OHW and NYH populations under water stress, but not in the IBM population (Fig. 9 and Supplementary Table S1). In the field, water stress reduced shoot biomass by 37% and reduced yield by 49% (Fig. 9). In high-CN genotypes, the reduction of shoot biomass ranged from 41 to 49%, and the yield reduction was from 53 to 68%. In low-CN genotypes, the percentage reduction of shoot biomass varied from 19 to 36%, and yield was reduced from 28 to 50%. Shoot biomass under water stress was closely associated with CN in both mesocosms (*r* ^2^=0.51, *P*=0.04) and field (*r* ^2^=0.82, *P*=0.0013; Fig. 10). Under water stress, low-CN lines had significantly less leaf area than high-CN lines (Supplementary Tables S6 and S7). Finally, relative yield (water stress yield divided by unstressed yield) was strongly negatively related to CN (*r* ^2^=0.89, *P*=0.0003; Fig. 10).

## Discussion

Our results support the hypothesis that low CN improves water acquisition under water stress by increasing deep soil exploration (Table 1 and Figs 5 and 6). Under water stress, maize lines with low CN had a smaller portion of plant C balance devoted to root respiration (Fig. 4), greater rooting depth (Figs 5 and 6), greater water acquisition from deep soil strata (Table 1 and Fig. 8), better plant water status (Fig. 3), and therefore greater stomatal conductance and photosynthesis (Fig. 3), growth, and yield (Figs 9 and 10) than high-CN genotypes.

We obtained comparable results from water stress treatments in greenhouse mesocosms and field rainout shelters. Mesocosms are simplified, controlled environments, yet allow the direct measurement of total intact root respiration and permit detailed physiological analyses as entire root systems can be excavated. The field environment includes environmental factors such as soil temperature regimes, natural rainfall, soil biota, and soil physical properties that may affect results. Comparable results from both environments indicate that potentially confounding factors are not likely to be important.

We employed near-isophenic contrasts among RILs to explore the physiological utility of CN under water stress. RILs are ideally suited to the physiological analysis of phenotypes controlled by multiple alleles in unknown ways, as is the case with CN (Burton *et al.*, 2014). No quantitative trait loci for CN were found in the three RIL populations employed in this study despite moderate heritabilities (Burton *et al.*, 2014). We selected RILs varying in CN yet otherwise phenotypically similar (Supplementary Tables S6–S8). RILs share genetic backgrounds (i.e. RILS within a population share parents). The fact that our results were consistent in field and mesocosm environments with different sets of RILs indicates that the utility of CN for deep water capture doesn’t depend on the specific genotypic context.

CN is an important determinant of soil resource capture (Lynch, 2013). Previous studies from field and greenhouse environments report that CN varies among maize genotypes from five to >70 (Bayuelo-jiménez *et al.*, 2011; Gaudin *et al.*, 2011; Trachsel *et al.*, 2011; Burton *et al.*, 2013). Our field CN ranged from 25 to 62 and falls in the medium to high range of phenotypic variation observed in maize. We propose that an intermediate CN may be ideal (Lynch, 2013). If the CN is too low, axial roots may be too dispersed to sufficiently acquire soil resources, such plants may be susceptible to lodging (Hetz *et al.*, 1996), and such phenotypes may be at risk of root loss due to herbivores and pathogens, especially in low-input agroecosystems. However, if the CN is too large, crown roots may compete with each other for soil resources, as well as for internal metabolic resources, resulting in reduced elongation and wasted effort under stress conditions (Lynch, 2013). Therefore, the optimal range of CN is likely to depend on soil type and the severity of biotic and abiotic stresses (Saengwilai *et al.*, 2014*b*).

It has been postulated that optimal CN can interact with other traits enhancing deep soil exploration, such as steep root growth angle (Trachsel *et al.*, 2013) and reduced lateral branching (Zhan and Lynch, 2015; Zhan *et al.*, 2015), and may synergistically improve resource acquisition under drought and suboptimal availability of mobile nutrients (Lynch, 2013). Using the functional–structural plant model *SimRoot*, York *et al.* (2013) found that the synergistic effects of CN and root cortical aerenchyma on plant growth were greater than the additive effects by 32% at medium N and by 132% at medium phosphorus. More recently, using stepwise multiple linear regression analysis, York and Lynch (2015) found that the additive integration of several phenes (e.g. nodal root number, angle, and lateral root length density), though each with small effects, can explain almost 70% of the variation observed in shoot mass in low N soils. In the present study, low CN genotypes under WS had 30% less lateral branching of crown roots, and 17% steeper crown root angles compared with low CN genotypes under well-watered conditions (see Supplementary Table S8), suggesting that the combination of low CN, less lateral root branching and steeper root angles can be synergistic for water acquisition under drought.

IBM123 had an unusual CN phenotype. This genotype had relatively high CN in the field, yet had deep water acquisition (Figs 7 and 8). This may be due to the timing of root development in this genotype. In mesocosms at 35 DAP it had few crown roots (Fig. 1), whereas in the field at anthesis it had relatively more crown roots (Fig. 1). It is possible that a low CN phenotype early in vegetative growth afforded advantages for water capture.

Plants can modulate metabolic partitioning to optimize plant growth by balancing tradeoffs among roots (Krassovsky, 1926; Walk *et al.*, 2006; Rubio and Lynch, 2007; Saengwilai *et al.*, 2014*b*). Krassovsky (1926) found that the removal of nodal roots stimulates the growth and activity of seminal roots in wheat and barley. Walk *et al.* (2006) used *SimRoot* to model bean root systems with varying architecture and C availability, and found that increased carbon allocation to adventitious roots was related to decreased allocation to tap and basal roots, which affected total root length, soil exploration, and phosphorus acquisition under suboptimal phosphorus conditions. Removal of specific root classes led to a compensatory increases in the relative proportion of the remaining root classes (Rubio and Lynch, 2007). In the present study, high-CN genotypes had significantly more crown roots than low-CN genotypes in high-order (fourth and fifth in greenhouse and fourth, fifth and sixth in the field) nodes under water stress, while there was no significant difference in the first, second and third nodes (Fig. 2). In maize, the majority of axial roots in the root system are crown roots, contributing 60–80% biomass of roots. The diameter of crown roots of the third and subsequent nodes is larger than that of primary and seminal roots, and these roots are thus a greater sink for plant assimilates (Saengwilai *et al.*, 2014*b*). High-CN genotypes must maintain the growth and development of many crown roots, which would constrain the growth and elongation of crown roots and other root classes, resulting in shallower root systems compared with those of low-CN genotypes (Figs 5 and 6). In contrast, fewer crown roots would conserve internal plant resources by reducing intra-plant root competition, allowing remaining crown root axes to elongate more rapidly, thereby improving deep water capture, plant water status, and plant growth and yield under water stress (Figs 3–6, 9 and 10 and Table 1).

Maintenance of soil water capture is an important element of the adaptation of annual crops to water deficit. Annual crops cannot accelerate phenology, go dormant, shed leaves, etc. without substantially reducing yield (Nord and Lynch, 2009). During the development of terminal drought, soil dries from the top of the profile exposing the upper part of the root system to water stress, while deeper roots may still be able to access deeper soil water (Saradadevi *et al.*, 2014). Therefore, deep-rooted cultivars have a yield advantage under drought (Lorens *et al.*, 1987; Zhu *et al.*, 2010; Chimungu *et al.*, 2014*a*, *b*; Zhan and Lynch, 2015). Indeed, there is a growing body of evidence that in many drought environments, rooting depth is positively related to soil exploration and greater acquisition of water from deep soil strata, leading to greater yield in various crops (Sponchiado *et al.*, 1989; Ho *et al.*, 2005; Manschadi *et al.*, 2006; Lopes and Reynolds, 2010; Zhu *et al.*, 2010; Wasson *et al.*, 2012; Uga *et al.*, 2013; Chimungu *et al.*, 2014*a*, *b*). Therefore, increased rooting depth is one of the most important ways to improve plant fitness under water scarcity (Lynch and Wojciechowski, 2015). We observed greater crown root density in deep soil of low CN phenotypes than high CN phenotypes in both mesocosms and the field (Fig. 5 and Supplementary Table S4) as well as strong negative relationships between CN and crown rooting depth and root length density in deep soil (Fig. 6). Marginally significant correlations were observed between crown root number with rooting depth and root length density in the deep soil for primary and seminal roots (see Supplementary Fig. S5), emphasizing the importance of crown roots for water acquisition.

Several abiotic (i.e. nitrogen and water) and biotic factors may affect nodal root development. Previous studies have shown that nodal root number and the number of nodes vary among genotypes, and both are decreased by low nitrogen availability (Gaudin *et al.*, 2011; Gao *et al.*, 2015; York and Lynch, 2015). Consistent with these results, Saengwilai *et al.* (2014*b*) also found that reduced CN by N stress was attributable to fewer crown root nodes and fewer roots per node. Several studies in cereals (i.e. wheat, sorghum and millet) showed that drought stress reduced fine root length density and inhibited new nodal root growth (Rostamza *et al.*, 2013; Steinemann *et al.*, 2015). In the current study, the number of seminal roots (data not shown) and the first three nodes of crown roots were not much affected by water stress (Fig. 2). However, with prolonged drought stress, the development of nodal roots and the number of the root nodes was inhibited (Fig. 2). The inhibition of nodal root development by water stress could be driven by reduced internal carbohydrate availability and/or by signaling mechanisms such as ABA (Westgate and Boyer, 1985; Deak and Malamy, 2005). Our results suggest that reduced formation of nodal roots is a positive adaptation of plants to water stress.

Our results and those of Saengwilai *et al.* (2014*b*) suggest that reduced formation of axial roots under N and water stress is adaptive. A recent study found that over the past century commercial maize lines have developed root phenotypes, including reduced CN, that are more efficient at N capture in high density stands (York *et al.*, 2015). We propose that the evolution of maize from wild plants to ancient polycultures (Postma and Lynch, 2012; Zhang *et al.*, 2014) to increasing high density monocultures has progressively reduced the optimum CN, by reducing interspecific root competition, increasing intraspecific root competition, and decreasing root loss to herbivores and pathogens.

The rhizoeconomic paradigm indicates that plant fitness under water- and nutrient-limiting conditions is influenced by the balance of the benefits and the costs of root traits as direct metabolic costs, tradeoffs and risks (Lynch and Ho, 2005; de Kroon and Mommer, 2006; Lynch, 2014). A number of studies have shown that the metabolic costs of soil exploration by root systems are substantial (Lambers *et al.*, 2002; Zhu *et al.*, 2005). All else being equal, a plant that is able to acquire a limiting soil resource at reduced metabolic cost will have superior productivity because it will retain more metabolic resources available for further resource acquisition, growth and reproduction (Lynch, 2014). Accumulating evidence indicates plants can increase root depth by reducing the metabolic cost of soil exploration through anatomical traits such as decreased root cortical cell file number and increased cortical cell size (Chimungu *et al.*, 2014*a*, *b*), through increased formation of root cortical aerenchyma (Zhu *et al.*, 2010; Postma and Lynch, 2011; Saengwilai *et al.*, 2014*a*; Chimungu *et al.*, 2015), and through architectural phenes, such as decreased CN (Saengwilai *et al.*, 2014*a*) and lateral root branching density (Postma *et al.*, 2014; Zhan *et al.*, 2015; Zhan and Lynch, 2015). These phenotypes increase soil exploration by allocating more C to subsoil foraging for water and nutrient acquisition (Lynch, 2014; Lynch and Wojciechowski, 2015). It appears that the low-CN phenotype adjusts biomass allocation to more efficiently allocate root foraging to deep soil strata, although total root C investment in roots was similar in both low- and high-CN genotypes (Figs 4, 5 and 10). In this way, under water stress maize lines with low CN had a smaller portion of plant C gain devoted to root respiration than maize lines with high CN, resulting in greater net C gain, shoot biomass and yield in the low CN phenotype (Figs 3, 4 and 9); it is noteworthy that specific root respiration (pmol CO_2_ cm^−1^ root length s^−1^) was similar in low- and high-CN genotypes (Supplementary Fig. S3 and Supplementary Table S1).

In the current study, natural variation in the isotopic signature of soil water (Dawson and Pate, 1996; Durand *et al.*, 2007; Jobbágy *et al.*, 2011) was used to provide insight into the depth of water acquisition by contrasting genotypes (Table1 and Fig. 7). Stem water δ^18^O signatures showed that the low CN phenotype had lighter isotope signatures and therefore greater dependency on deep soil water than the high CN phenotype (Table 1). The difference in the depths of root water acquisition between the low CN and high CN genotypes could be attributed to their rooting depth (Table 1 and Figs 7 and 8).

The steep, cheap and deep (SCD) ideotype proposes that reduced CN will improve the capture of water and N by increasing rooting depth (Lynch, 2013). Our results support the inclusion of reduced crown root number as an element of the SCD ideotype (Lynch, 2013) for enhanced water (Table 1 and Figs 7 and 8) and N acquisition (Saengwilai *et al.*, 2014*a*) when those resources restrain plant growth. The SCD ideotype is applicable to both water and N capture, since both of these resources are often localized in deep soil strata under stress conditions. We suggest that reduced CN would improve water capture in other Poaceae species. The root system architecture of sorghum is similar to that of maize (Lynch, 2013), so the optimal CN concept may be applicable to sorghum. Other cereals such as wheat, rice, barley, and oats have the same basic root structure as maize and should also benefit from an optimum CN, although greater density of nodal roots and reduced whorl development in tillering species may change the relationship of nodal root occupancy and resource capture. This merits investigation.

Many traditional metrics of root phenotypes are actually phene aggregates with low heritability, showing high plasticity in response to soil conditions (de Dorlodot *et al.*, 2007; York *et al.*, 2013; Burton *et al.*, 2014; Lynch, 2014). Genotypic differences in CN have been reported in maize (Trachsel *et al.*, 2011; Burton *et al.*, 2014; Saengwilai *et al.*, 2014*a*). Previous studies demonstrate that CN is a heritable trait (Hetz *et al.*, 1996; Burton *et al.*, 2014) and genes affecting CN expression have been identified in maize (Hetz *et al.*, 1996; Taramino *et al.*, 2007; Muthreich *et al.*, 2013) and rice (Redillas *et al.*, 2012; Uga *et al.*, 2013; Gao *et al.*, 2014). Our results support the hypothesis that low CN phenotypes have increased rooting depth, resulting in greater water acquisition from deep soil strata, improved net carbon gain, and improved growth and yield under water stress. Crown root number merits investigation as a potential element to improve drought tolerance in crop breeding programs.

## Supplementary data

Supplementary data are available at *JXB* online.

Figure S1. Soil water content from 0–140cm depth in well-watered and water-stressed conditions at 35 days after planting in greenhouse mesocosms.

Figure S2. Field soil volumetric water content at 10, 30, and 50cm depths in well-watered and water-stressed treatments.

Figure S3. Specific root respiration at 35 DAP in greenhouse mesocosms under water-stressed and well-watered conditions.

Figure S4. Correlations between crown root number and rooting depth from 80–140cm soil depths of maize at 35 DAP in greenhouse mesocosms and from 40–60cm soil depth at anthesis in the field under well-watered conditions.

Figure S5. Correlations between crown root number and rooting depth and root length density of primary roots and seminal roots from 80–140cm soil depths of maize at 35 DAP in greenhouse mesocosms under water-stressed conditions.

Table S1. Summary of analysis of variance for crown root number, shoot dry weight, leaf relative water content, leaf photosynthesis, leaf stomatal conductance, canopy photosynthesis, total root respiration and specific root respiration at 35 days after planting in greenhouse mesocosms as influenced by soil moisture regimes, genotypes, crown root phenotypes and their interactions.

Table S2. Analysis of the effect of plasticity of IBM123 at field and NYH51 in the greenhouse in water stress conditions.

Table S3. Summary of analysis of variance for crown root number, shoot dry weight, leaf relative water content, leaf photosynthesis, leaf stomatal conductance at anthesis, yield at physiological maturity in the field as influenced by soil moisture regimes, genotypes, crown root phenotypes and their interactions.

Table S4. Means of total root surface for the whole soil profile and total root surface in deep soil layers with contrasting crown root number of phenotypes at anthesis in the rainout shelters and in the greenhouse.

Table S5. Means of average root diameter in the top soil 0–10cm and in deep soil layers with contrasting crown root number of phenotypes at anthesis in the rainout shelters and in the greenhouse.

Table S6. Means of leaf area and leaf number measured for eight maize recombinant inbred lines with contrasting crown root number at anthesis in the rainout shelters at Pennsylvania.

Table S7. Means of leaf area and leaf number measured for eight maize recombinant inbred lines with contrasting crown root number at 35 days after planting in greenhouse mesocosms.

Table S8. Means of lateral root branching density of crown and crown root angles measured for eight maize recombinant inbred lines with contrasting crown root number at anthesis in the rainout shelters at Pennsylvania.

Supplementary Data
