# Supplementary material for: Reduced crown root number improves water acquisition under water deficit stress in maize (Zea mays L.)
Source: J Exp Bot. 2016 Jul 8;67(15):4545–57. doi: 10.1093/jxb/erw243 (PMC4973737; doi:10.1093/jxb/erw243)
Supplement: Supplementary Data [file supp_67_15_4545__index.html]

Supplementary Data 

# Reduced crown root number improves water acquisition under water deficit stress in maize (*Zea mays* L.)

## Supplementary Data

Data files

- supplementary\_figure\_S1.TIF - Supplementary Data
- supplementary\_figure\_S2.TIF - Supplementary Data
- supplementary\_figure\_S3.TIF - Supplementary Data
- supplementary\_figure\_S4.TIF - Supplementary Data
- supplementary\_figure\_S5\_tables\_S1\_S8.pdf - Supplementary Data
