# Supplementary figures and images for: Reduced crown root number improves water acquisition under water deficit stress in maize (Zea mays L.)
Source: J Exp Bot. 2016 Jul 8;67(15):4545–57. doi: 10.1093/jxb/erw243 (PMC4973737; doi:10.1093/jxb/erw243)

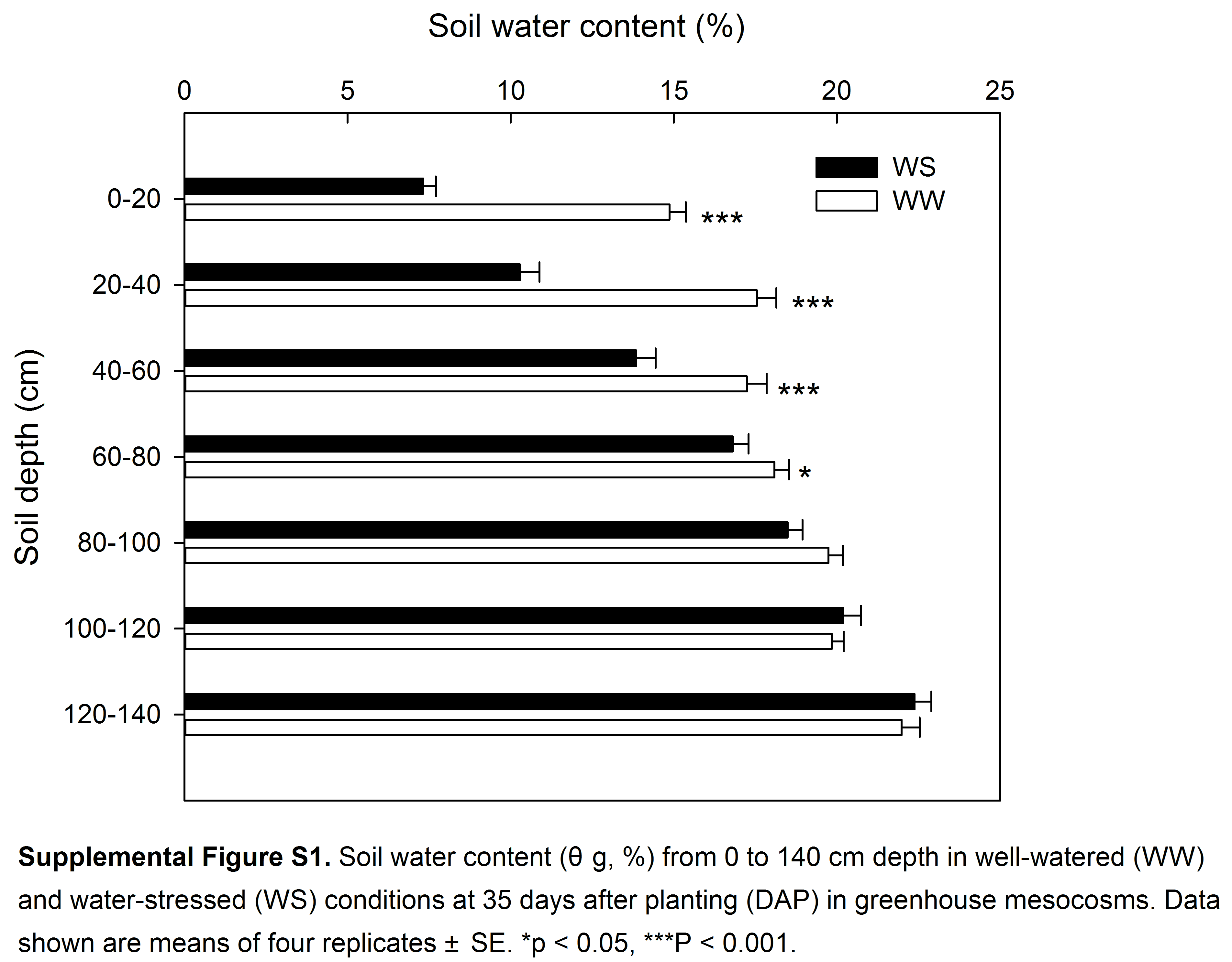

Supplement: Supplementary Data [file supp_erw243_supplementary_figure_S1.tif]

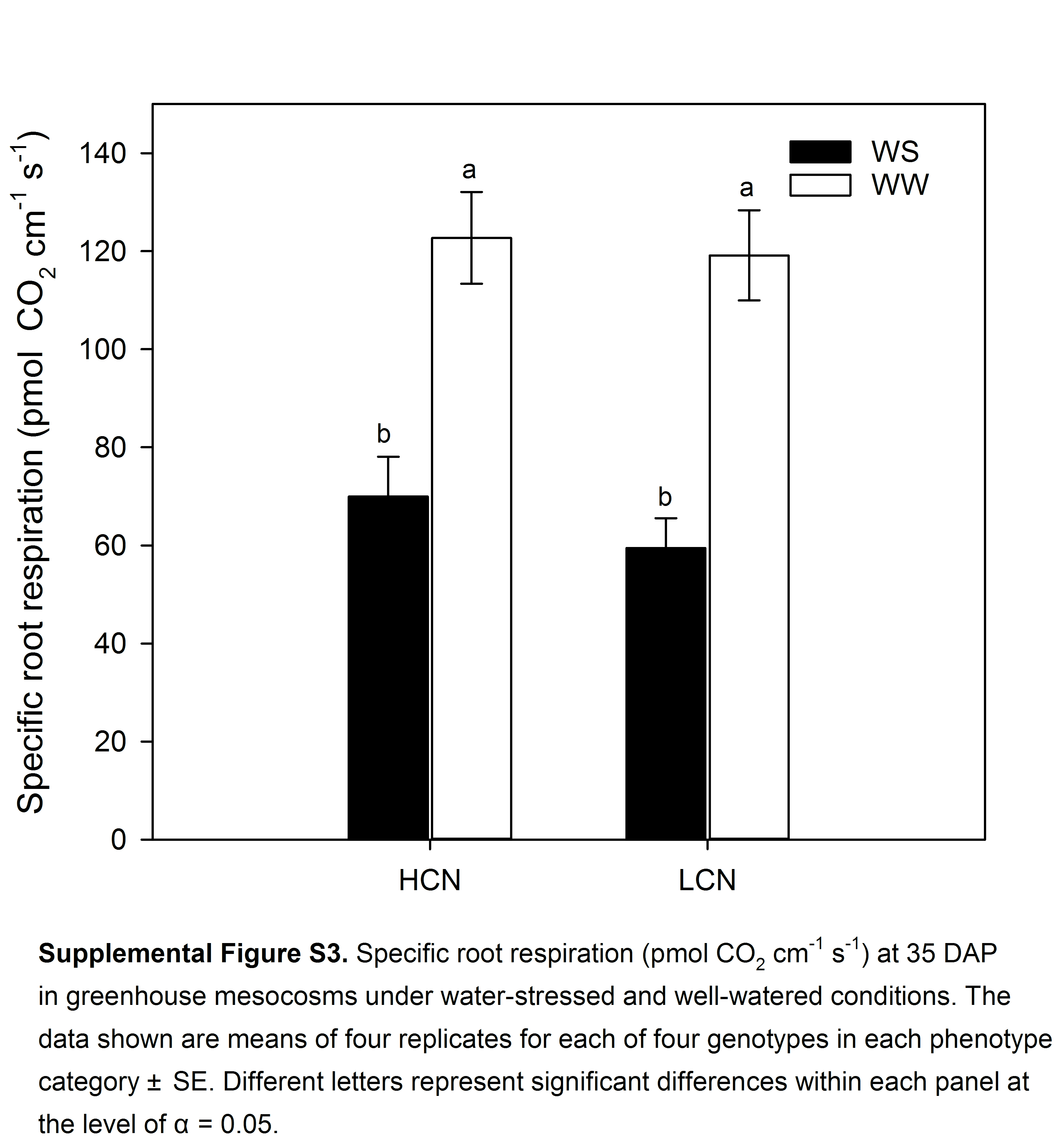

Supplement: Supplementary Data [file supp_erw243_supplementary_figure_S3.tif]

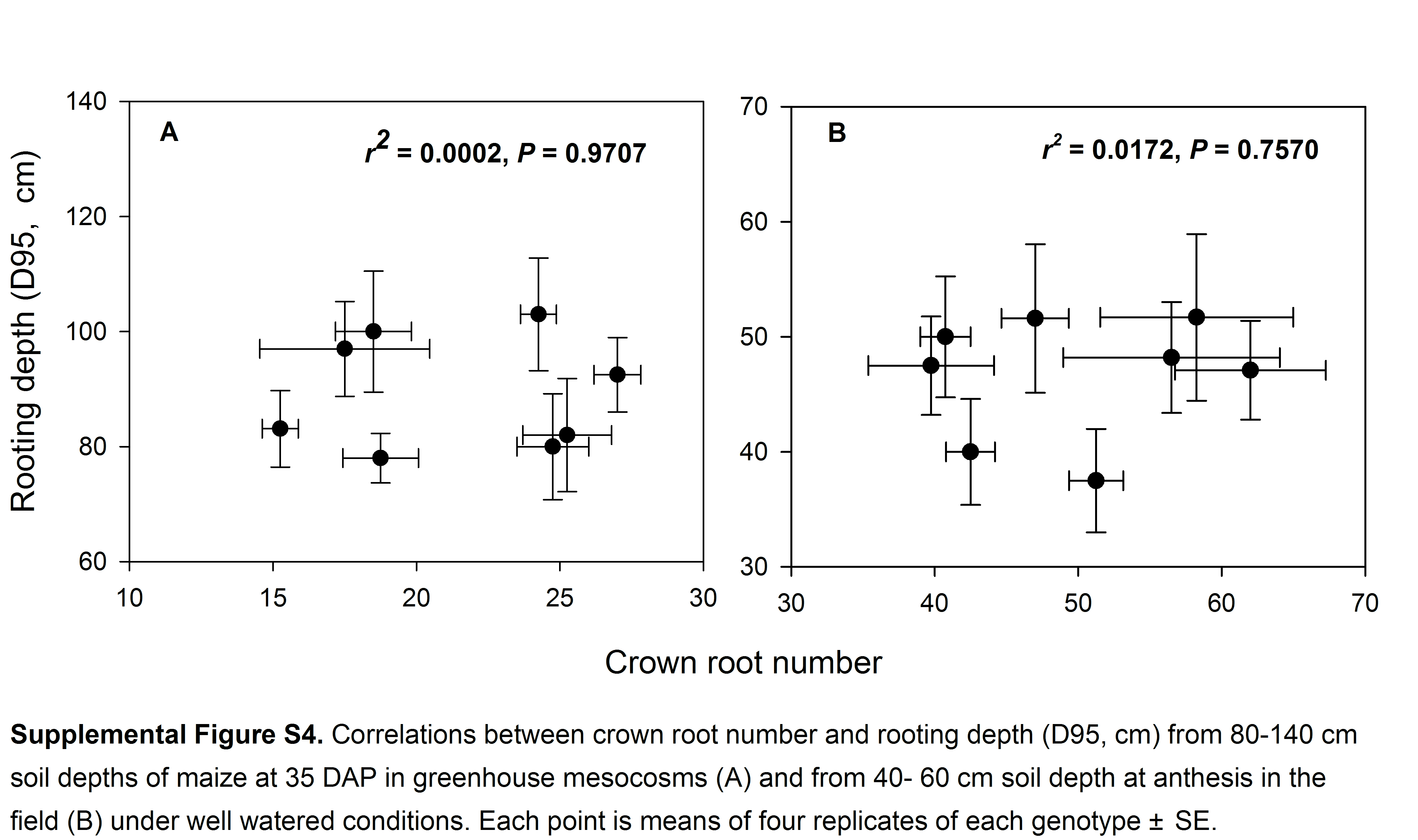

Supplement: Supplementary Data [file supp_erw243_supplementary_figure_S4.tif]
