# Supplementary material for: Reduced crown root number improves water acquisition under water deficit stress in maize (Zea mays L.)
Source: J Exp Bot. 2016 Jul 8;67(15):4545–57. doi: 10.1093/jxb/erw243 (PMC4973737; doi:10.1093/jxb/erw243)
Supplement: Supplementary Data [file supp_erw243_supplementary_figure_S5_tables_S1_S8.pdf]

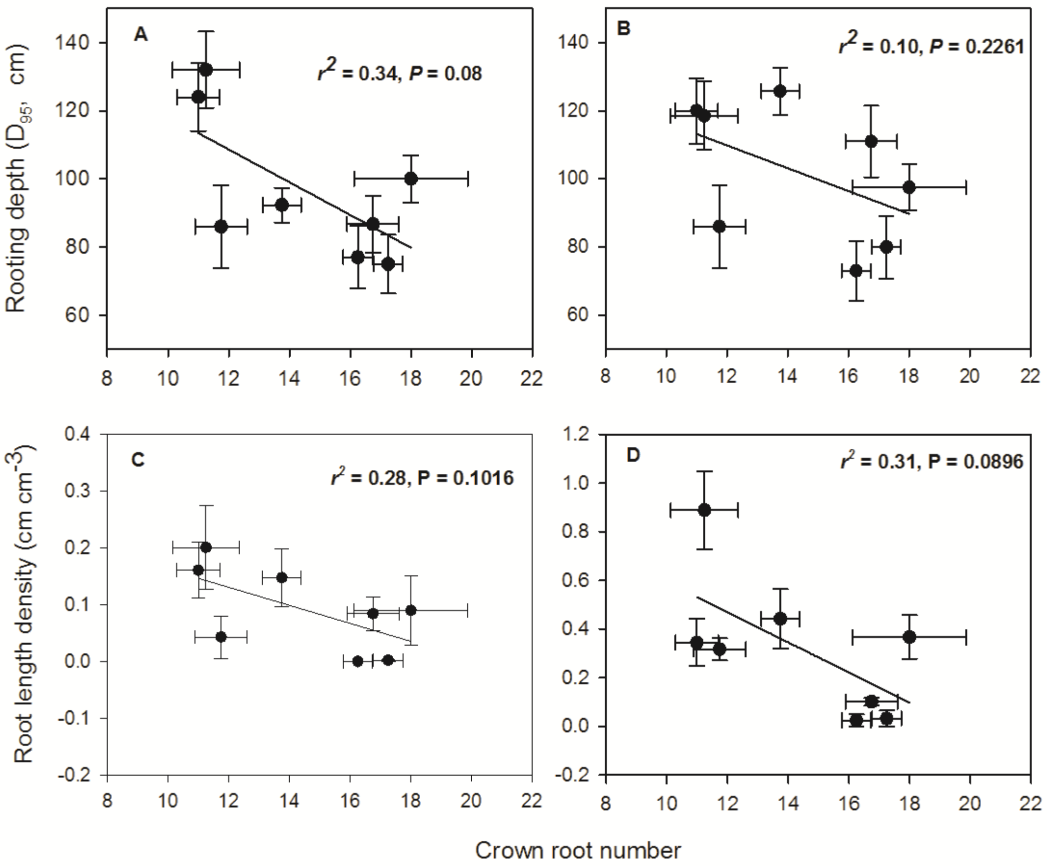

Supplemental Figure S5. Correlations between crown root number and rooting depth (D95, cm) and root length density (cm cm^{-3}) of primary roots (PR) (A and C) and seminal roots (SR) (B and D) from 80-140 cm soil depths of maize at 35 DAP in greenhouse mesocosms under water-stressed conditions. Each point is means of four replicates of each genotype ± SE.

Supplemental Table 1. Summary of analysis of variance for crown root number (CN), shoot dry weight (SDW), leaf relative water content (LRWC, %), leaf photosynthesis (LPn, μmol CO_2 m^{-2} s^{-1}), leaf stomatal conductance (LSC, mol H_2O m^{-2} s^{-1}), canopy photosynthesis (CPn, μmol CO_2 s^{-1} $plant^{-1}$), total root respiration (TRS, μmol CO_2 s^{-1} $plant^{-1}$) and specific root respiration (SRR, pmol CO_2 cm^{-1} root length s^{-1}) at 35 days after planting (DAP) in greenhouse mesocosms as influenced by soil moisture regimes (treatment (T)), genotypes (G), crown root phenotypes (P) and their interactions. The associated F-values and probabilities (NS, not significant; *, P<0.1; **, P<0.01; ***, P<0.001) are shown.

Source of variation	CN	SDW	LRWC	LPn	LSC	CPn	TRS	SRR
Treatment (T)	121.19***	277.12***	508.03***	569.17***	429.55***	119.89***	201.98***	50524***
Genotype (G)	16.57***	5.27***	3.05*	25.59***	4.69***	1.83NS	1.01NS	2039.73*
G × T	0.94NS	3.28**	5.13***	15.48***	3.73**	2.83*	0.32NS	142.29NS
phenotypes (P)	107.03***	2.96*	17.71***	49.33***	11.76***	8.19***	0.92NS	791.07NS
P × T	4.61*	7.78*	34.71***	36.81***	20.72***	21.15***	0.76NS	190.61NS

Supplemental Table 2. Analysis of the effect of plasticity of IBM123 at field and NYH51 in the greenhouse in water stress (WS) conditions A) All replications of genotype IBM123 and NYH51 classified as low crown root number (LCN); B) All replications of IBM123 and NYH51classified as high crown root number (HCN); C) IBM123 and NYH51 removed from dataset.

	CO_2 assimilation rate (µmol CO_2 m^{-2} s^{-1})		Stomatal conductance (mmol H_2O m^{-2} s^{-1})		Leaf relative water content (%)		Net canopy assimilation (µmol CO_2 s^{-1} $plant^{-1}$)		Total root respiration (µmol CO_2 s^{-1} $plant^{-1}$)		Crown root number		D_{95} (cm)	
	**LCN**	**HCN**	**LCN**	**HCN**	**LCN**	**HCN**	**LCN**	**HCN**	**LCN**	**HCN**	**LCN**	**HCN**	**LCN**	**HCN**
IBM123														
**A**	25.3	19.7	0.24	0.17	86.3	77.2	-	-	-	-	30.8	39.2	52.2	36.8
**B**	25.7	20.6	0.25	0.18	87.0	78.5	-	-	-	-	28.7	38.6	53.0	39.4
**C**	25.7	19.7	0.25	0.17	87.0	77.2	-	-	-	-	28.7	39.2	53.0	36.8
NYH51														
**A**	16.0	11.0	0.11	0.07	83.3	77.7	378.9	236.0	41.2	41.6	11.9	17.1	111.5	85.5
**B**	16.3	11.8	0.11	0.08	83.7	78.6	385.7	260.6	43.7	40.0	11.3	16.4	112.4	89.5
**C**	16.3	11.0	0.11	0.07	83.7	77.7	385.7	236.0	43.7	41.6	11.3	17.1	112.4	85.5

Supplemental Table 3. Summary of analysis of variance for crown root number (CN), shoot dry weight (SDW), leaf relative water content (LRWC, %), leaf photosynthesis (LPn, μmol CO_2 m^{-2} s^{-1}), leaf stomatal conductance (LSC, mol H_2O m^{-2} s^{-1}) at anthesis, yield at physiological maturity in the field as influenced by soil moisture regimes (treatment (T)), genotypes (G), crown root phenotypes (P) and their interactions. The associated F-values and probabilities (NS, not significant; *, P<0.05; **, P<0.01; ***, P<0.001) are shown.

Source of variation	CN	SDW	LRWC	LPn	LSC	Yield
Treatment (T)	68.09***	111.31***	316.07***	363.98***	363.06***	187.19***
Genotype (G)	7.66***	3.36**	6.03***	4.65***	1.99NS	3.63**
G × T	0.76NS	2.61*	4.45**	4.84**	2.91**	2.36*
phenotypes (P)	38.04***	3.88*	39.78***	18.53***	3.44*	6.80*
P × T	3.15NS	7.89**	28.55***	28.7***	9.29***	9.41***

Supplemental Table 4. Means of total root surface (TRS, $cm^2 \pm SE$) for the whole soil profile and total root surface in deep soil layers (TRSD) with contrasting crown root number of phenotypes (HCN: high crown root number; LCN: low crown root number) at anthesis in the rainout shelters and in the greenhouse. Deep soil layers mean 40-60 cm in the rainout shelters, and 80-140 cm in the greenhouse. The different letters within the column in the same growing conditions are significantly different at the α = 0.05 level.

**Data sources**	**Phenotypes**	**WW**		**WS**	
		TRS	TRSD	TRS	TRSD
**Field**	**HCN**	94.06±7.046 a	18.51±6.68 a	85.03±6.86 a	8.34±1.98 a
	**LCN**	111.13±8.03 a	25.49±8.29 a	81.83±5.94 a	19.66±3.66 b
**Greenhouse**	**HCN**	2816±89 a	795±56 a	1713±71 a	398±52 a
	**LCN**	2717±79 a	703±47 a	1884±75 a	703±47 b

Supplemental Table 5. Means of average root diameter (ARD, mm ± SE) in the top soil 0-10 cm and in deep soil layers (ARD) with contrasting crown root number of phenotypes (HCN: high crown root number; LCN: low crown root number) at anthesis in the rainout shelters and in the greenhouse. Deep soil layers means 40-60 cm in the rainout shelters, and 80-140 cm in the greenhouse. The different letters within the column in the same growing conditions are significantly different at the α = 0.05 level.

**Data source**	**Phenotypes**	**WW**		**WS**	
		ARD 0-10	ARDD	ARD 0-10	ARDD
**Field**	**HCN**	0.42±0.03a	0.49±0.34a	0.37±0.02a	0.49±0.05a
	**LCN**	0.42±0.02a	0.50±0.03a	0.37±0.02a	0.47±0.04a
**Greenhouse**	**HCN**	0.32±0.02a	0.46±0.03a	0.30±0.02a	0.44±0.02a
	**LCN**	0.33±0.02a	0.44±0.03a	0.29±0.01a	0.46±0.02a

Supplemental Table 6. Means of leaf area (m^2 $plant^{-1}$) and leaf number ± SE measured for eight maize recombinant inbred lines (RILs) with contrasting crown root number (HCN: high crown root number; LCN: low crown root number) at anthesis in the rainout shelters in Pennsylvania. The different letters within the column are significantly different at the α = 0.05 level.

Classification based on CN	RILs	WW		WS	
		Leaf area	Leaf number	Leaf area	Leaf number
**HCN**	**IBM009**	**0.38 ± 0.03**	**9.63 ± 0.24**	**0.16 ± 0.01**	**6.90 ± 0.20**
	**OHW170**	**0.37 ± 0.03**	**9.75 ± 0.52**	**0.18 ± 0.02**	**7.38 ± 0.24**
	**NYH47**	**0.35 ± 0.03**	**10.38 ±0.24**	**0.17 ± 0.03**	**7.63 ± 0.31**
	**NYH57**	**0.41 ± 0.04**	**10.25 ± 0.66**	**0.21 ± 0.01**	**7.95 ± 0.24**
**LCN**	**IBM123**	**0.37 ± 0.03**	**9.50 ± 0.35**	**0.20 ± 0.02**	**7.01 ± 0.24**
	**OHW74**	**0.41 ± 0.01**	**10.25 ± 0.25**	**0.30 ± 0.02**	**8.88 ± 0.69**
	**NYH51**	**0.36 ± 0.01**	**9.38 ± 0.31**	**0.26 ± 0.03**	**8.58 ± 0.31**
	**NYH224**	**0.36 ± 0.02**	**10.63 ± 0.38**	**0.28 ± 0.03**	**8.79 ± 0.20**
**Mean**	**HCN**	**0.38 ± 0.01a**	**10.25 ± 0.27a**	**0.18 ± 0.02a**	**7.47 ± 0.16a**
	**LCN**	**0.37 ± 0.01a**	**9.93 ± 0.20a**	**0.26 ± 0.01b**	**8.34 ± 0.25b**

Supplemental Table 7. Means of leaf area (dm^2 plant^{-1}) and leaf number ± SE measured for eight maize recombinant inbred lines (RILs) with contrasting crown root number (HCN: high crown root number; LCN: low crown root number) at 35 days after planting (DAP) in greenhouse mesocosms. The different letters within the column are significantly different at the α = 0.05 level.

Classification based on CN	RILs	WW		WS	
		Leaf area	Leaf number	Leaf area	Leaf number
**HCN**	**IBM009**	**13.90 ± 1.51**	**7.50 ± 0.29**	**6.57 ± 0.50**	**6.25 ± 0.63**
	**OHW170**	**18.86 ± 0.90**	**8.50 ± 0.29**	**7.01 ± 0.66**	**6.85 ± 0.48**
	**NYH47**	**14.84 ± 0.43**	**8.50 ± 0.65**	**5.53 ± 0.45**	**6.50 ± 0.29**
	**NYH57**	**18.36 ± 1.83**	**9.50 ± 0.29**	**5.88 ± 0.46**	**6.25 ± 0.63**
**LCN**	**IBM123**	**14.32 ± 0.86**	**7.75 ± 0.25**	**6.62 ± 0.86**	**6.25 ± 0.25**
	**OHW74**	**17.05 ± 2.07**	**9.00 ± 0.41**	**8.88 ± 1.61**	**7.25 ± 0.25**
	**NYH51**	**14.12 ± 0.73**	**8.25 ± 0.48**	**6.89 ± 1.04**	**7.00 ± 0.41**
	**NYH224**	**18.75 ± 1.45**	**8.75 ± 0.48**	**7.19 ± 0.40**	**6.50 ± 0.29**
**Mean**	**HCN**	**16.49 ± 0.80a**	**8.50 ± 0.26a**	**6.25 ± 0.28a**	**6.44 ± 0.24a**
	**LCN**	**16.06 ± 079a**	**8.44 ± 0.22a**	**7.40 ± 0.53b**	**6.75 ± 0.17a**

Supplemental Table 8. Means of lateral root branching density (LRBD, branch cm^{-1}) of crown and crown root angles (CRA, °) ± SE measured for eight maize recombinant inbred lines (RILs) with contrasting crown root number (HCN: high crown root number; LCN: low crown root number) at anthesis in the rainout shelters in Pennsylvania. The different letters within the column are significantly different at the at α = 0.05 level.

Classification based on CN	RILs	WW		WS	
		LRBD	CRA	LRBD	CRA
**HCN**	**IBM009**	11.43±0.28 a	48.65±1.13 a	7.93±0.45 a	55.95±0.82 ab
	**OHW170**	10.50±0.34 a	47.38±1.05 a	7.40±0.93 a	54.98±1.51 b
	**NYH47**	9.85±0.42 a	49.38±1.04 a	7.43±0.53 a	56.60±0.88 ab
	**NYH57**	11.00±0.54 a	47.30±0.91 a	8.33±0.77 a	55.77±1.52 ab
**LCN**	**IBM123**	10.50±0.39 a	48.85±1.12 a	7.08±0.37 a	59.63±1.89 a
	**OHW74**	11.35±0.70 a	50.98±1.47 a	7.40±0.21 a	56.25±1.22 ab
	**NYH51**	9.40±0.74 a	48.95±1.04 a	7.25±0.54 a	56.08±0.96 ab
	**NYH224**	10.43±0.59 a	46.90±0.75 a	7.30±0.43 a	56.30±1.77 ab
**Mean**	**HCN**	10.69±0.24 a	48.18±0.52 a	7.77±0.33 a	55.83±0.57 ab
	**LCN**	10.41±0.33 a	48.92±0.63 a	7.26±0.19 a	57.06±0.78 ab
